# EdeepSADPr: an extensive deep-learning architecture for prediction of the *in situ* crosstalks of serine phosphorylation and ADP-ribosylation

**DOI:** 10.3389/fcell.2023.1149535

**Published:** 2023-04-28

**Authors:** Haoqiang Jiang, Shipeng Shang, Yutong Sha, Lin Zhang, Ningning He, Lei Li

**Affiliations:** ^1^ College of Basic Medicine, Qingdao University, Qingdao, China; ^2^ Sino Genomics Technology Co., Ltd., Qingdao, China; ^3^ College of Computer Science and Technology, Qingdao University, Qingdao, China; ^4^ Faculty of Biomedical and Rehabilitation Engineering, University of Health and Rehabilitation Sciences, Qingdao, China

**Keywords:** proteomics, machine learning and AI, post-translational modification (PTM), phosphorylation, prediction model, ADP-ribosylation, bioinforamtics

## Abstract

The *in situ* post-translational modification (PTM) crosstalk refers to the interactions between different types of PTMs that occur on the same residue site of a protein. The crosstalk sites generally have different characteristics from those with the single PTM type. Studies targeting the latter’s features have been widely conducted, while studies on the former’s characteristics are rare. For example, the characteristics of serine phosphorylation (pS) and serine ADP-ribosylation (SADPr) have been investigated, whereas those of their *in situ* crosstalks (pSADPr) are unknown. In this study, we collected 3,250 human pSADPr, 7,520 SADPr, 151,227 pS and 80,096 unmodified serine sites and explored the features of the pSADPr sites. We found that the characteristics of pSADPr sites are more similar to those of SADPr compared to pS or unmodified serine sites. Moreover, the crosstalk sites are likely to be phosphorylated by some kinase families (e.g., AGC, CAMK, STE and TKL) rather than others (e.g., CK1 and CMGC). Additionally, we constructed three classifiers to predict pSADPr sites from the pS dataset, the SADPr dataset and the protein sequences separately. We built and evaluated five deep-learning classifiers in ten-fold cross-validation and independent test datasets. We also used the classifiers as base classifiers to develop a few stacking-based ensemble classifiers to improve performance. The best classifiers had the AUC values of 0.700, 0.914 and 0.954 for recognizing pSADPr sites from the SADPr, pS and unmodified serine sites, respectively. The lowest prediction accuracy was achieved by separating pSADPr and SADPr sites, which is consistent with the observation that pSADPr’s characteristics are more similar to those of SADPr than the rest. Finally, we developed an online tool for extensively predicting human pSADPr sites based on the CNN_OH_ classifier, dubbed EdeepSADPr. It is freely available through http://edeepsadpr.bioinfogo.org/. We expect our investigation will promote a comprehensive understanding of crosstalks.

## 1 Introduction

The *in situ* post-translational modification (PTM) crosstalk refers to the interactions between different types of PTMs that occur on the same residue site of a protein. Different PTM types on the same site have different effects on the activity, stability, localization, and interactions of the modified protein (Yang and Gregoire, 2006; Hunter, 2007; Swaney et al., 2013; Xu et al., 2018). The crosstalk sites generally have different characteristics from those with the single PTM type; Nevertheless, the former is rarely investigated compared to the latter. This study focused on the crosstalk between serine phosphorylation (pS) and ADP-ribosylation (SADPr). Serine phosphorylation, catalyzed by hundreds of kinases, plays a regulatory role in the cell cycle, growth, apoptosis, and signal transduction (Zolnierowicz and Bollen, 2000). Serine ADP-ribosylation, catalyzed by over twenty ADP-ribosyltransferases (Luscher et al., 2018), regulates many cellular processes, including chromatin organization, epigenetic transcription regulation, cell differentiation and cytoplasm stress response (Nowak et al., 2020; Brustel et al., 2022). Both serine modifications can co-occur on the same residue on a competitive basis as the *in situ* PTM crosstalk (dubbed pSADPr). This crosstalk represents a significantly high degree of overlap, similar to the site-specific crosstalk between lysine acetylation and ubiquitylation (Larsen et al., 2018). Identification of PTM crosstalk sites has emerged to be an intriguing topic and attracted much attention, relevant works of which have been ongoing before our study (Peng et al., 2014; Venne et al., 2014; Xu et al., 2021). For example, the classifier mUSP was developed to predict *in situ* crosstalk sites of ubiquitylation and SUMOylation (Xu et al., 2021). Nevertheless, the *in situ* crosstalk of serine phosphorylation and ADP-ribosylation has not been investigated. Although a few *in silico* classifiers have been developed for predicting pS and SADPr sites (Luo et al., 2019; Sha et al., 2021), the classifier for predicting pSADPr sites is unavailable.

**FIGURE 1:**
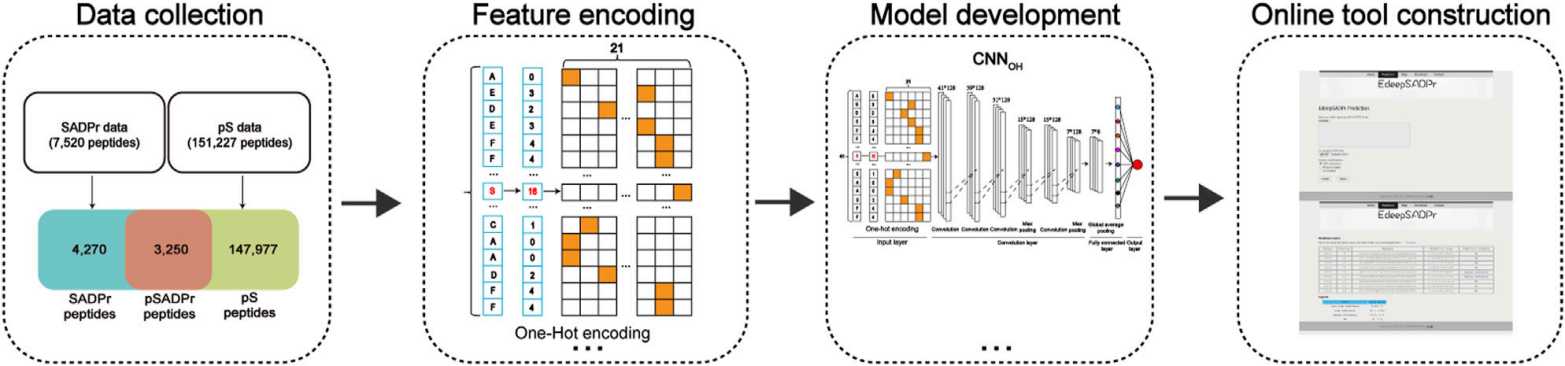
Flowchart of the model construction.

Figure 1 showed the overview map of this study. This study collected 3,250 human pSADPr, 151,227 pS, 7,520 SADPr and 80,096 unmodified serine sites. Accordingly, we investigated the characteristics of pSADPr and constructed classifiers to predict pSADPr sites. We found that pSADPr’s characteristics are more similar to those of SADPr than pS and unmodified serine sites. We also found that pSADPr sites were preferred to be phosphorylated by four subfamilies of serine kinases (i.e., AGC, CAMK, STE and TKL). Moreover, we built and evaluated five deep-learning classifiers in ten-fold cross-validation and independent test datasets. We also developed a few advanced stacking-based ensemble classifiers. The best classifiers had the AUC values of 0.700, 0.914 and 0.954 for recognizing pSADPr sites from the SADPr, pS and unmodified serine sites. Finally, we developed an online tool for extensively predicting human pSADPr sites, dubbed EdeepSADPr. It is freely available through http://edeepsadpr.bioinfogo.org/. We anticipate that accurate prediction by EdeepSADPr will facilitate the discovery of new pSADPr sites and promote the understanding of their functional characteristics.

## 2 Materials and methods

### 2.1 Data collection and preprocessing

**FIGURE 2:**
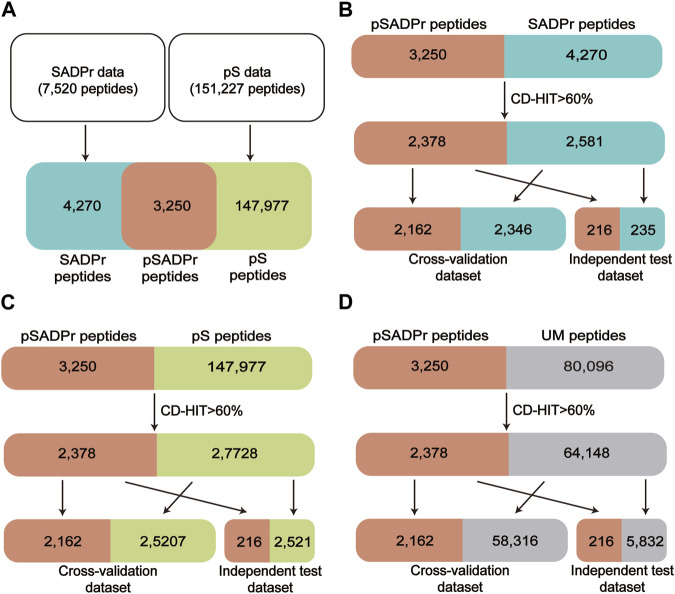
Schematic diagram of data collection and preprocessing for human pSADPr datasets. Construction of the pSADPr, pS and SADPr datasets **(A)**. The construction and preprocessing of the pSADPr-SADPr dataset **(B)**, the pSADPr-pS dataset **(C)** and the pSADPr-UM dataset **(D)**. UM stands for unmodified serine.

Figure 2 shows the procedure of dataset construction and preprocessing. 7,520 human SADPr sites with high confidence (i.e., ADPr peptides with Andromeda scores >40 and localization probability >0.75) were collected from the literature (Larsen et al., 2018; Hendriks et al., 2019; Buch-Larsen et al., 2020; Nowak et al., 2020) (Figure 2A). 151,227 human pS sites were obtained from the database PhosphositePlus (Hornbeck et al., 2012) and the literature (Luo et al., 2019) (Figure 2A). We compared both datasets and found 3,250 pSADPr peptides, 147,977 pS peptides, and 4,270 SADPr peptides. We also collected 80,096 unmodified serine (UM) sites after removing modified serine sites (i.e., pSADPr, SADPr and pS) from the reported dataset (Luo et al., 2019).

Each serine site of the above datasets was represented by a 41-residue-long sequence segment with the serine at the center (Sha et al., 2021). CD-HIT (Li and GodzikCd-hit, 2006; Huang et al., 2010) was applied to eliminate the homologous peptides by setting the threshold to 60% sequence identity, which is valuable for avoiding overestimation. Specifically, we combined the pSADPr peptides with SADPr peptides, pS peptides, and UM peptides, respectively, and clustered them using CD-HIT. Accordingly, we obtained 4,959 clusters, 30,106 clusters and 66,526 clusters. We selected one sequence randomly from each cluster according to the criterion: One pSADPr peptide was selected if it was included in the cluster; otherwise, one of the other peptides was selected. After that, 2,378 pSADPr, 2,581 SADPr, 27,728 pS and 64,148 UM peptides were collected (Figures 2B–D). Furthermore, each of the three datasets was divided into 11 groups, where ten groups were used as a cross-validation dataset, and the rest group was considered an independent test dataset (Figures 2B–D). It should be noted that if the central serine residue is located near the N or C terminus of the protein sequence, the complement symbol “_” was added to the input sequences at the affected terminus to ensure the length was maintained. All these data are available at http://edeepsadpr.bioinfogo.org/.

### 2.2 Feature encoding schemes

We selected five encoding features representing the input peptides for the model construction. They included the One-Hot encoding (OH) (Wang D. et al., 2020), the Enhanced Amino Acid Composition Encoding (EAAC) (Chen et al., 2018), the Enhanced Grouped Amino Acids Content encoding (EGAAC) (Chen et al., 2018), the ZSCALE Encoding (ZSCALE) and the Word Embedding (WE).

#### 2.2.1 One-hot (OH) encoding

In the One-hot coding, the 20 amino acids and complement symbol “_” are encoded into a 21-dimensional binary vector. In the vector corresponding to an amino acid, the element related to the amino acid is marked as 1 and others are marked as 0. For example, “A” is represented by “100000000000000000000” and “V” is represented by “0100000000000000000000.”

#### 2.2.2 ZSCALE encoding

In ZSCALE encoding, every amino acid type is characterized by five physicochemical descriptor variables (Chen et al., 2012; Zhang et al., 2020). Therefore, each input sequence is represented as a vector of 205 (=41 × 5) dimensions. The filling character “_” is encoded as a 5-dimensional zero vector.

#### 2.2.3 Word embedding (WE) encoding

Word embedding (Ge and Moh, 2018) relies on the numerical encoding approach (Lyu et al., 2020), which maps each type of amino acid residue to an integer. After the NUM encoding, each integer is mapped to a predefined five-dimension word vector. Therefore, each sequence is encoded as a vector of 205 (= 41 × 5) items.

#### 2.2.4 Enhanced amino acid composition (EAAC) encoding

In EAAC encoding, the frequency of each amino acid from the N-terminal to the C-terminal within a fixed sliding window size (the default length being 5) is calculated (Lyu et al., 2020). Therefore, each peptide sequence is encoded as a vector of 740 = ((41–5 + 1) × 20) items.

#### 2.2.5 Enhanced grouped amino acids content (EGAAC) encoding

The EGAAC encoding is developed based on grouped amino acid content (GAAC) characteristics (Wei et al., 2021). In the GAAC encoding, the 20 amino acid types are divided into five groups according to their physical and chemical properties (G1: GAVLMI, FYW, G3: KRH, G4: DE, and G5: STCPNQ). In the EGAAC encoding, the GAAC value is calculated from N-terminal to C-terminal within a fixed sliding window (the default length being 5).

### 2.3 The architecture of deep-learning classifiers

**FIGURE 3:**
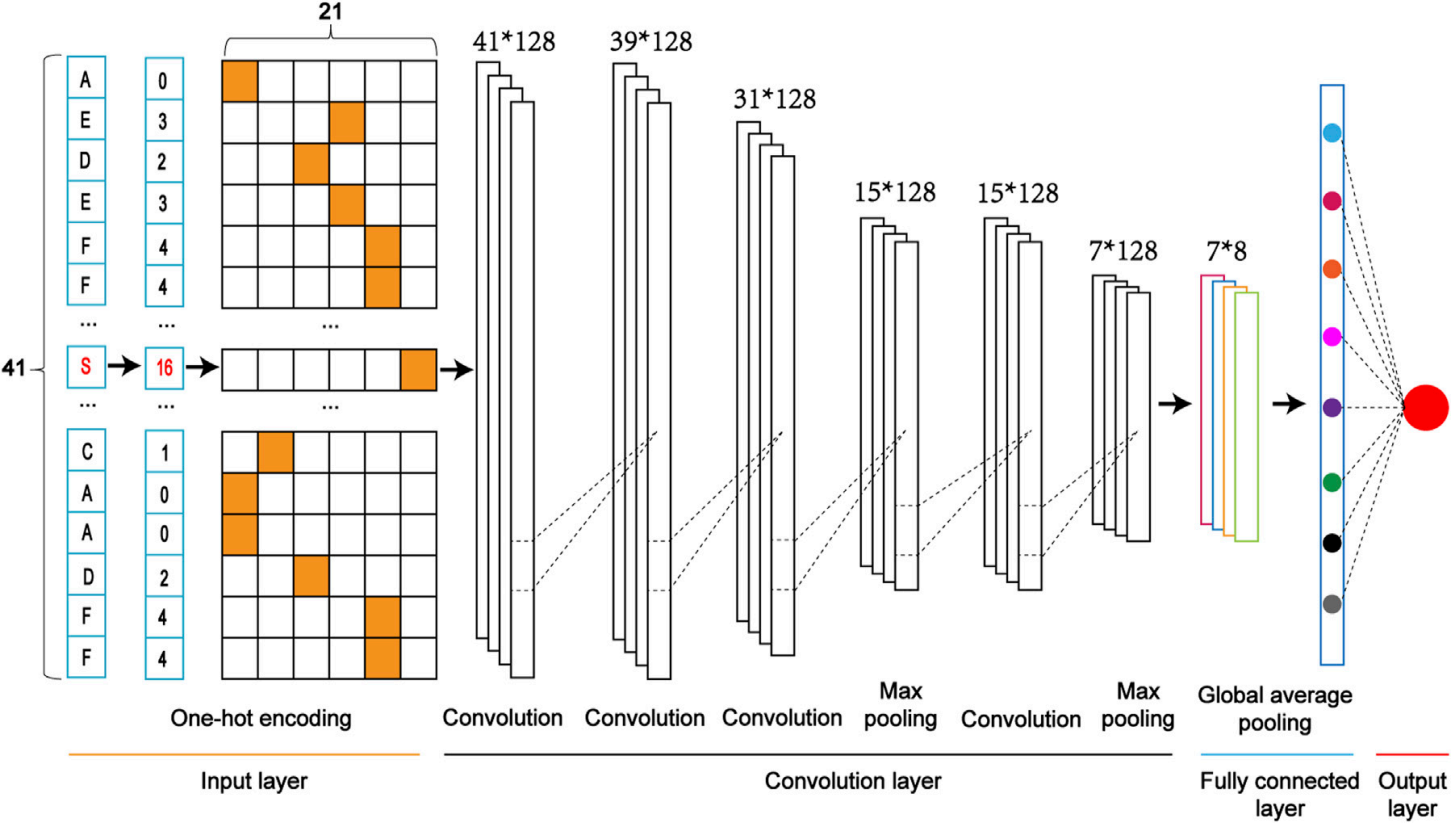
The architecture of a one-dimensional convolutional neural network with the One-Hot encoding approach (i.e., CNN_OH_).

We constructed five classifiers based on Convolutional Neural Network (CNN). They included the model combined with the One-Hot Encoding (CNN_OH_), the model with the Word Embedding Encoding (CNN_WE_), the model with the ZSCALE Encoding (CNN_ZSCALE_), the model with the EAAC encoding (CNN_EAAC_) and the model with the EGAAC encoding (CNN_EGAAC_). We took the CNN Model with the One-Hot encoding (CNN_OH_) as an example to demonstrate the architecture (Figure 3).

(1) Input layer. Each sequence is converted into a feature vector with One-Hot encoding.
(2) The convolution layer. It contains two convolution sublayers followed by two sequentially connected blocks. Each block includes a convolution sublayer and a max pooling sublayer. There are 128 convolution kernels with the sizes of 1 and 3 for the first and second convolution sublayers, respectively. A dropout layer with a rate of 0.7 follows each convolution kernel to prevent potential overfitting. In these two blocks, there were 128 convolution kernels with a size of 9 and 10 for these two convolution sublayers of two blocks, respectively; the parameter pool_size of the max-pooling sublayer was set as 2; the dropout rate was set to 0.5. The rectified linear unit (ReLU) is considered the activation function.
(3) Fully connected layer. It contains a dense sublayer with 128 neurons without flattening and a global average pooling sublayer to calculate and output an average value.
(4) Output layer: This layer contains a single neuron, activated by a sigmoid function, to output the probability score (within the range from 0 to 1), indicating the likelihood of the crosstalk. If the probability score of an input sequence is greater than a specified threshold, the central serine in the sequence is predicted as a crosstalk site.

### 2.4 Performance evaluation

Several statistical measures were used to evaluate prediction performance, including sensitivity (SN), specificity (SP), overall accuracy (ACC), Matthew correlation coefficient (MCC) and the area under the receiver operating characteristic (ROC) curve (AUC). The definitions of SN, SP, ACC, and MCC are given as follows:

$$SN=\frac{TP}{TP+FN}$$

$$SP=\frac{TN}{TN+FP}$$

$$ACC=\frac{TP+TN}{TP+FP+TN+FN}$$

$$MCC=\frac{TP\times TN-TN\times FP}{\sqrt{\left(TP+FN\right)\times \left(TN+FP\right)\times \left(TP+FP\right)\times \left(TN+FN\right)}}$$

In the above formulas, TP, TN, FP, and FN are the number of true positives, true negatives, false positives, and true negatives, respectively.

## 3 Results and discussion

### 3.1 Construction and functional investigation of the pSADPr datasets

We created three datasets for constructing classifiers to predict pSADPr sites (Figure 2). The first dataset was the pSADPr-SADPr dataset, containing pSADPr and SADPr peptides. The related model was used to recognize pSADPr sites from known SADPr sites (Figure 2B). The second was the pSADPr-pS dataset, including pSADPr and pS peptides (Figure 2C). The third was the pSADPr-UM dataset, containing pSADPr and UM peptides (Figure 2D). Because the vast majority of serine residues are unmodified in the human proteome, the model based on the third dataset was expected to recognize pSADPr sites from the human proteome (Figure 2D). Each of the three datasets contained two parts: cross-validation and independent test datasets (Figures 2B–D).

**FIGURE 4:**
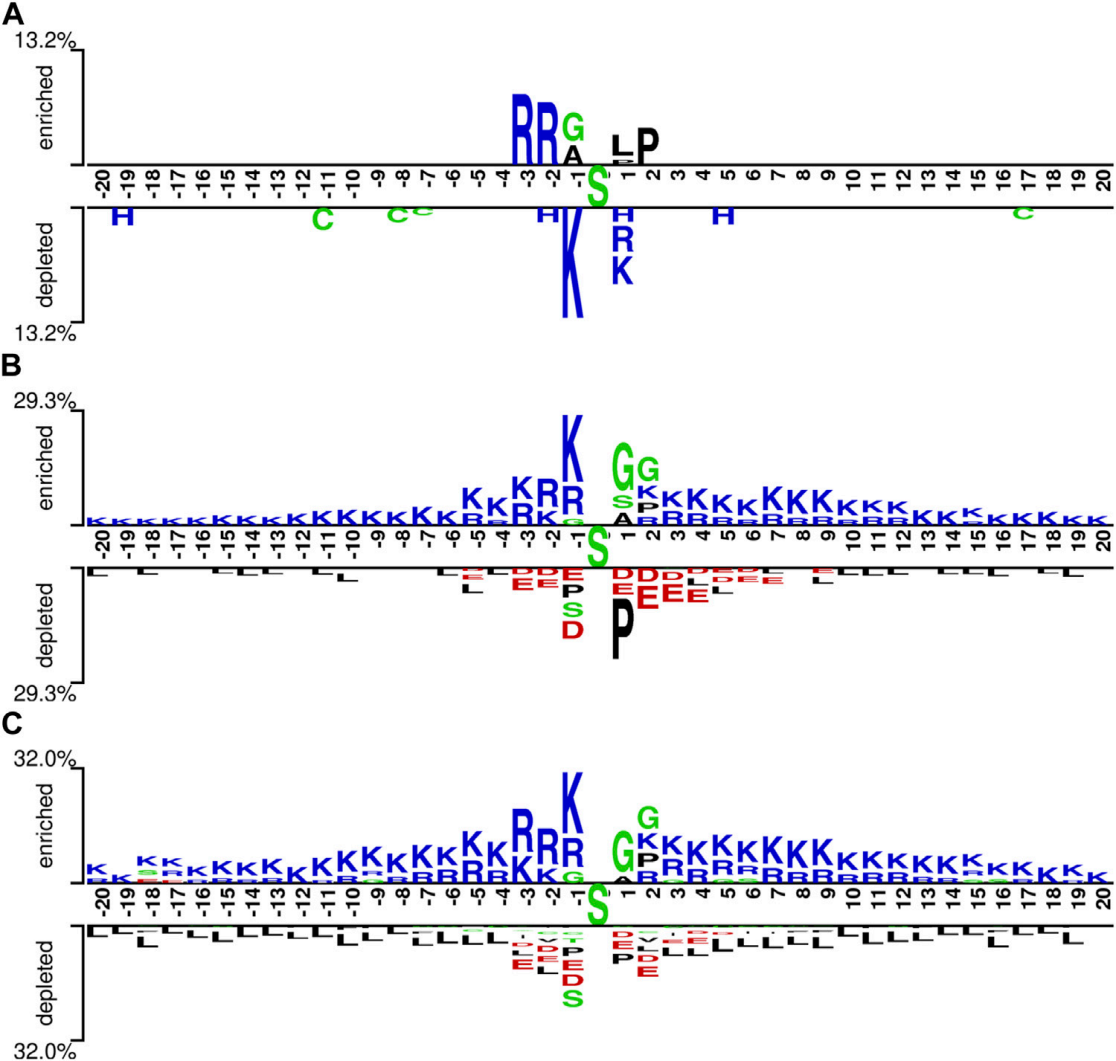
Sequence pattern surrounding the pSADPr sites. Enriched and depleted residues flanking the central pSADPr sites were shown for the pSADPr-SADPr dataset **(A)**, the pSADPr-pS dataset **(B)**, and the pSADPr-UM dataset **(C)** (*p* < 0.05, *t*-test with Bonferroni correction). The patterns were generated using the Two-Sample-Logo program (Vacic et al., 2006).

We explored the characteristics of the pSADPr crosstalks by comparing pSADPr-containing and other peptides in the three datasets through the Two-Sample-Logo program (Vacic et al., 2006). For the pSADPr-SADPr dataset, the amino acid R was significantly enriched at positions −2 and −3 (*i.e.*, P-2 and P-3), whereas K was depleted at P-1 (Figure 4A). For the rest datasets, the pSADPr crosstalks showed similar characteristics (Figures 4B, C). Specifically, K was enriched entirely except P+1 and G was enriched at P1 and P2; D and E were depleted at P-3 to P+5 and L was depleted entirely. The maximum enriched/depleted value (29.3%) for the pSADPr-pS dataset was similar to that (32.0%) for the pSADPr-UM dataset, and both were more than twice as large as that (13.2%) for the pSADPr-SADPr dataset (Figure 4). It indicates that the differences between pSADPr and SADPr sites are smaller than those between pSADPr and pS/UM sites. In other words, it is easy to distinguish pSADPr sites from pS/UM sites, compared to recognizing pSADPr sites from SADPr sites.

**FIGURE 5:**
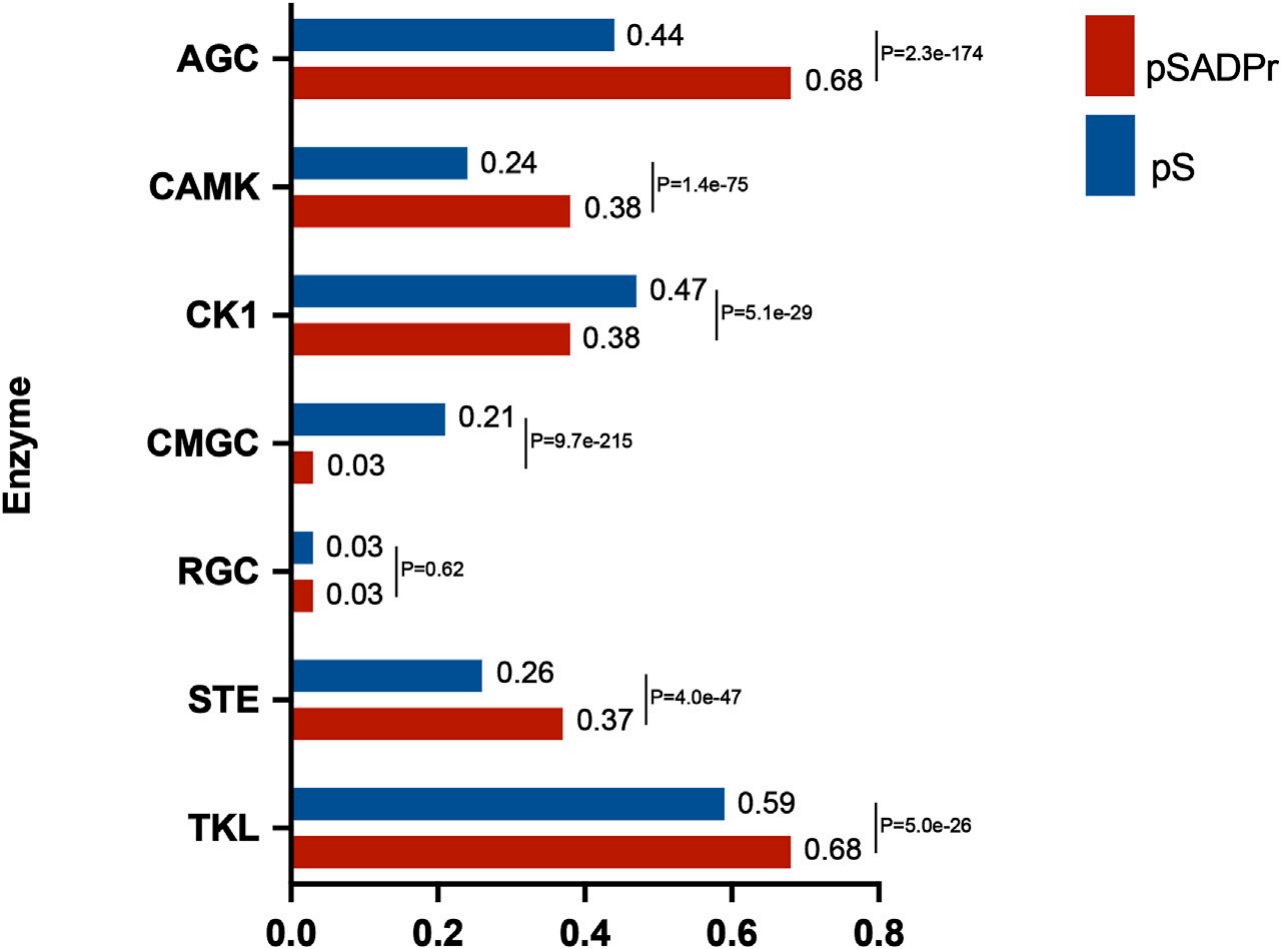
Enrichment analysis of human pSADPr sites as the substrates of serine kinase subfamilies predicted by GPS (Wang C. et al., 2020). Human pS sites were used as the background. *p*-value was calculated using the hyper-geometric test.

The human serine kinase family contains a few subfamilies, each with its characteristics. We explored which subfamilies preferred phosphorylating the pSADPr sites. To perform this analysis, we used the human pS sites as the background and the pSADPr sites as the test dataset. We employed the GPS program (Wang C. et al., 2020) to predict pS sites for each subfamily from both datasets (Figure 5). We found that four subfamilies (i.e., AGC, CAMK, STE and TKL) tended to phosphorylate pSADPr sites (*p* < 5.0 × 10^−26^, hyper-geometric test). In comparison, two subfamilies (i.e., CK1 and CMGC) prefer not to phosphorylate pSADPr sites (*p* < 5.1 × 10^−29^, hyper-geometric test). For example, 68% of pSADPr sites could be phosphorylated by the AGC subfamily, whereas only 44% of pS sites are modified by this subfamily (*p* = 2.3 × 10^−174^, hyper-geometric test). This observation suggests that the pSADPr sites may be related to specific subfamilies of serine kinases.

**FIGURE 6:**
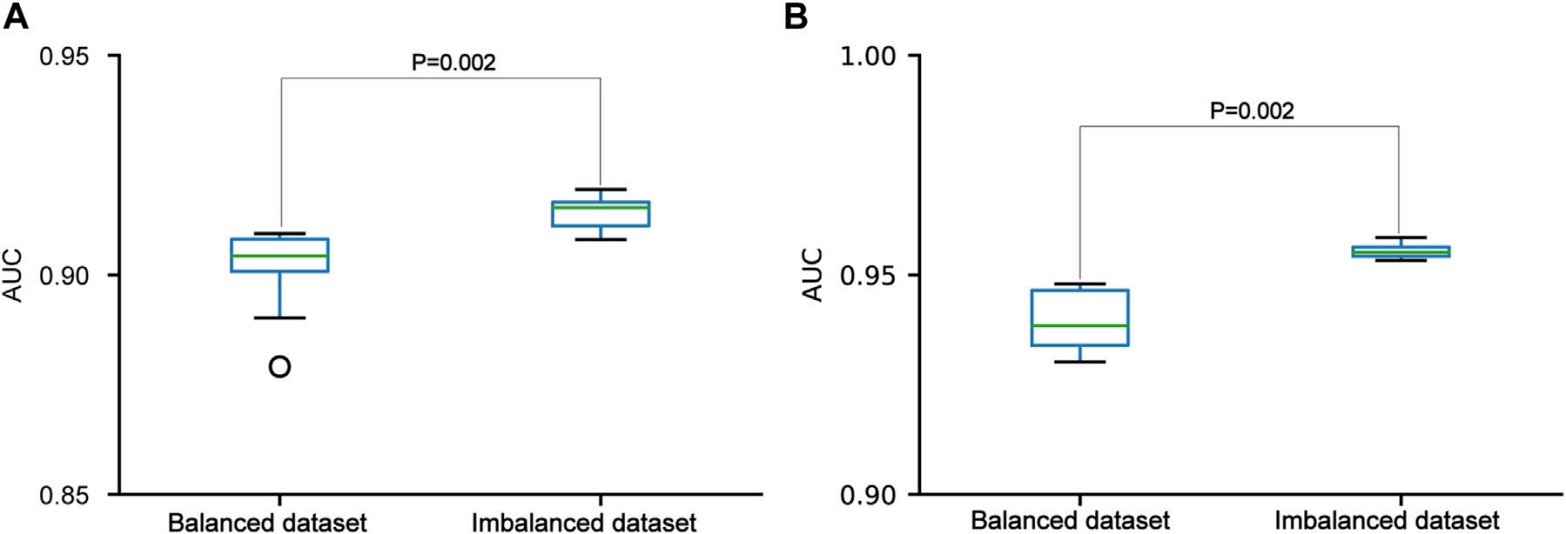
Performance comparisons between the CNN_OH_ models based on balanced and imbalanced datasets in the independent test dataset. The models were developed for the pSADPr-pS dataset **(A)** and the pSADPr-UM dataset **(B)**.

In the three datasets, the pSADPr-pS and pSADPr-UM datasets were imbalanced because the numbers of pS and UM peptides were far more than the number of pSADPr peptides (Figures 2C, D). To explore the effect of the imbalanced dataset on the predictor’s performance, we built the related balanced cross-validation dataset where the number (2,162) of randomly selected pS or UM peptides was the same as that of pSADPr peptides. We constructed the CNN_OH_ models related to the imbalanced and balanced datasets and evaluated their prediction performances in terms of the independent test. The CNN_OH_ model based on the imbalanced dataset had better performance than the counterpart constructed using the balanced dataset (*p* = 0.002 for both pSADPr-pS and pSADPr-UM datasets, Wilcoxon rank sum test; Figure 6). Therefore, we chose the imbalanced dataset for model construction.

### 3.2 Construction and evaluation of CNN-based classifiers

**FIGURE 7:**
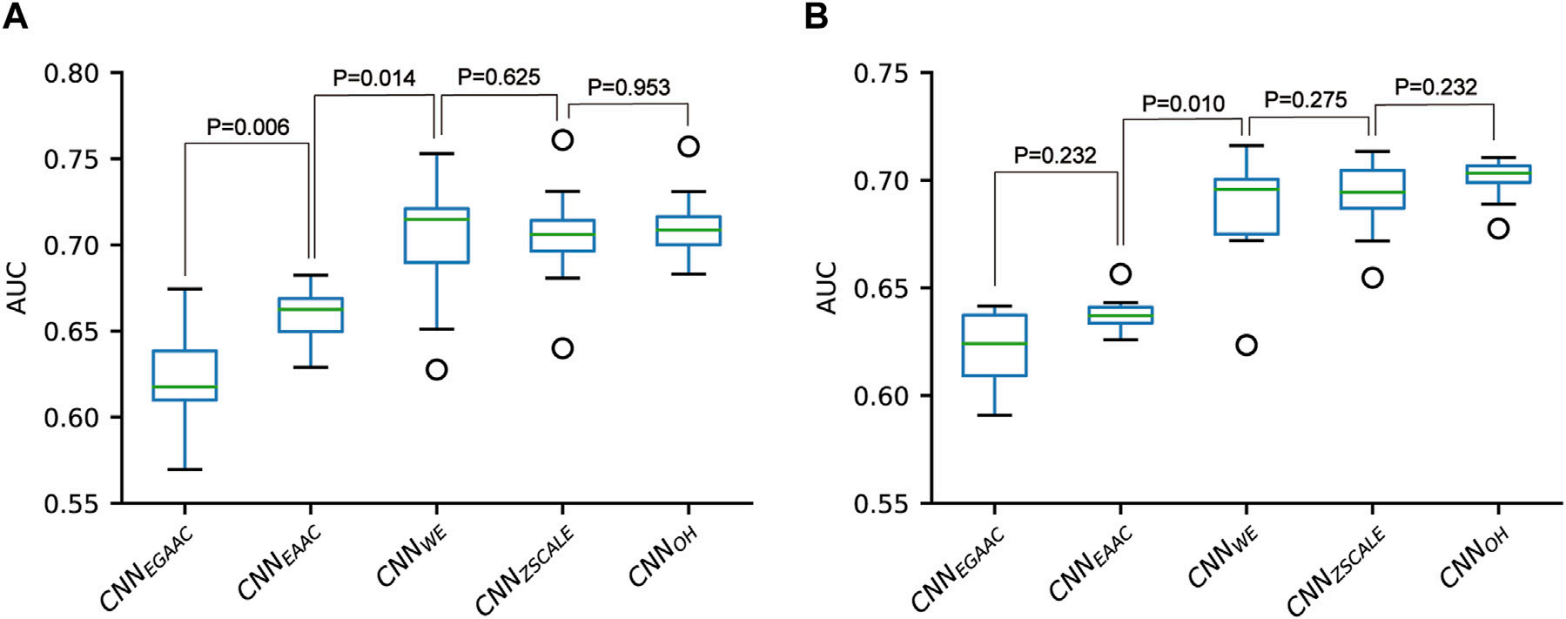
Performance comparison of CNN-based classifiers built for the pSADPr-SADPr dataset in ten-fold cross-validation **(A)** and independent test **(B)**.

We constructed five CNN classifiers (i.e., CNN_OH_, CNN_WE_, CNN_EAAC_, CNN_EGAAC_ and CNN_ZSCALE_) to recognize pSADPr sites from the three datasets and compared their prediction performances. Here, we used the pSADPr-SADPr dataset to demonstrate the process. Three out of the five classifiers (i.e., CNN_OH_, CNN_WE_ and CNN_ZSCALE_) showed similar performances and superiority over the rest two (i.e., CNN_EAAC_ and CNN_EGAAC_) in ten-fold cross-validation and independent test (Table 1; Figure 7 and Supplementary Figure S1). For instance, the CNN_OH_ model had an AUC value of 0.712, larger than that (0.659) of the CNN_EAAC_ model in the cross-validation. We repeated this analysis for the pSADPr-pS and pSADPr-UM datasets and made similar observations that the three classifiers had the best performances (Supplementary Tables S1, S2; Supplementary Figures S1–S5). Furthermore, we compared the classifiers’ performances for the three datasets. We found that the AUC values (0.921 and 0.953) of the CNN_OH_ classifiers for pSADPr-pS and pSADPr-UM datasets were significantly larger than that (0.712) for the pSADPr-SADPr dataset. These results were consistent with our observation that the differences between pSADPr and SADPr sites are smaller than those between pSADPr and pS/UM sites (Figure 4). Since the One-Hot feature is the simplest compared to the WE and ZSCALE features, we chose the CNN classifier with the One-Hot scheme as the representative of the three classifiers.

**TABLE 1:** Prediction performances of CNN-based classifiers for the pSADPr-SADPr dataset^a^.

Classifier	SN	SP	ACC	MCC	AUC
Ten-fold Cross-validation					
CNN_OH_	0.599 ± 0.031	0.694 ± 0.001	0.649 ± 0.016	0.294 ± 0.031	0.712 ± 0.020
CNN_ZSCALE_	0.598 ± 0.059	0.694 ± 0.001	0.649 ± 0.025	0.293 ± 0.058	0.705 ± 0.030
CNN_WE_	0.591 ± 0.089	0.694 ± 0.001	0.644 ± 0.044	0.285 ± 0.088	0.696 ± 0.043
CNN_EAAC_	0.523 ± 0.040	0.694 ± 0.001	0.611 ± 0.021	0.219 ± 0.040	0.659 ± 0.016
CNN_EGAAC_	0.488 ± 0.034	0.694 ± 0.001	0.595 ± 0.018	0.185 ± 0.034	0.621 ± 0.029
Independent test					
CNN_OH_	0.608 ± 0.034	0.694 ± 0.000	0.653 ± 0.016	0.303 ± 0.033	0.700 ± 0.010
CNN_ZSCALE_	0.583 ± 0.037	0.694 ± 0.000	0.641 ± 0.018	0.278 ± 0.036	0.692 ± 0.017
CNN_WE_	0.557 ± 0.058	0.694 ± 0.000	0.628 ± 0.028	0.253 ± 0.057	0.682 ± 0.022
CNN_EAAC_	0.500 ± 0.016	0.694 ± 0.000	0.601 ± 0.008	0.197 ± 0.016	0.637 ± 0.008
CNN_EGAAC_	0.488 ± 0.044	0.694 ± 0.000	0.595 ± 0.021	0.185 ± 0.043	0.621 ± 0.016

^a^ Ten models were constructed and evaluated in ten-fold cross-validation. Their average performance and standard deviation were separately calculated for the cross-validation and the independent test datasets.

### 3.3 Construction and evaluation of stacking ensemble learning classifiers

**FIGURE 8:**
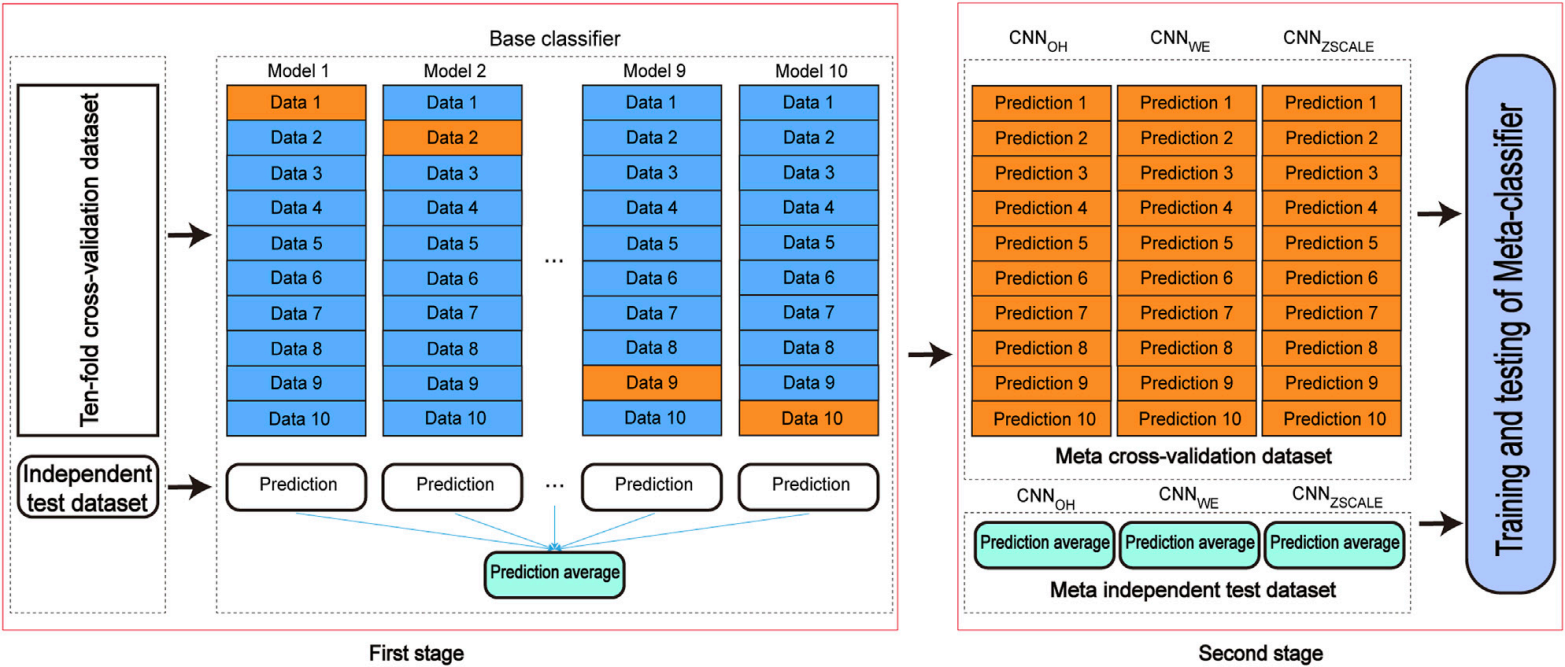
The architecture of the two-stage stacking ensemble classifier.

A stacking-based ensemble learning architecture is one of the ensemble techniques in which multiple learning models are integrated to produce one optimal predictive model, which performs better than the base models taken alone. In the stacking ensemble architecture, a meta-learner is trained to output a prediction based on the different base learner’s predictions. The stacking ensemble architecture has been used to improve the prediction performance in various bioinformatics applications (e.g., lysine acetylation site prediction) (Mishra et al., 2019; Zhang et al., 2021; Basith et al., 2022). Here, we introduced the two-stage stacking ensemble approach to improve the performance of the pSADPr site prediction (Figure 8). In the first stage, different CNN algorithms (e.g., CNN_OH_, CNN_WE_ and CNN_ZSCALE_) were selected to construct base classifiers. Specifically, ten base classifiers for each CNN algorithm were built and validated using the ten-fold cross-validation dataset. The base classifiers were then used for prediction in the independent test dataset, and their prediction results were averaged. Therefore, each CNN algorithm corresponds to the validation result and the averaged result for the independent test dataset. In the second stage, the validation and the averaged results were merged as a meta cross-validation dataset and a meta-independent test dataset, respectively (Figure 8). The former dataset was used to train and validate a meta-classifier, whereas the latter was employed to evaluate the meta-classifier’s performance. Here, we constructed the meta-classifier using the random forest algorithm (RF), which was optimized using the GridSearchCV package. The optimized parameters included max_depth as 8, max_features as “sqrt,” min_samples_leaf as 20, min_samples_split as 300 and n_estimators as 100.

**FIGURE 9:**
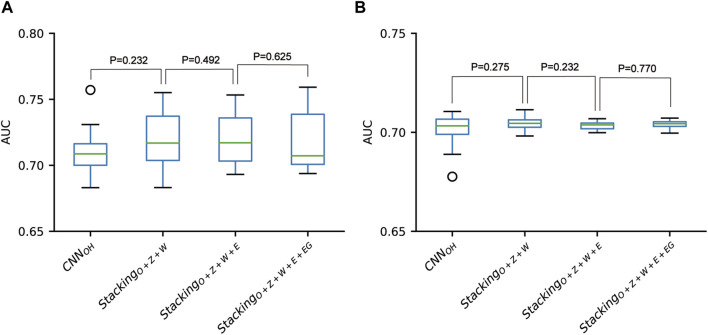
Performance comparison between CNN-based classifiers and the stacking-based ensemble classifiers for the pSADPr-SADPr dataset in the ten-fold cross-validation **(A)** and independent test **(B)**. *p* values were calculated using the two-sided Mann–Whitney U test.

According to the above analysis, the three classifiers (i.e., CNN_OH_, CNN_WE_ and CNN_ZSCALE_) had better performances than two other classifiers (i.e., CNN_EAAC_ and CNN_EGAAC_) for all three datasets. Based on the observation, we fused them as base classifiers to build the two-stage stacking ensemble approach with a good performance. We started with the fusion of the three best classifiers until we fused all the classifiers. The related stacking models included Stacking_O+Z+W_, Stacking_O+Z+W+E_ and Stacking_O+Z+W+E+EG_, where O stands for OH, Z for ZSCALE, W for WE, E for EAAC and, EG for EGAAC. For the pSADPr-SADPr dataset, the three stacking models showed similar performances in meta ten-fold cross-validation and independent test (Table 2; Figure 9 and Supplementary Figure S6). For instance, their average AUC/MCC values were around 0.719/0.313 in cross-validation (Table 2). The stacking models for the two other datasets (pSADPr-pS and pSADPr-UM) also performed similarly (Supplementary Figures S7–S10).

**TABLE 2:** Prediction performances of stacking ensemble classifiers for the pSADPr-SADPr dataset.

Classifier	SN	SP	ACC	MCC	AUC
Cross-validation					
CNN_O+Z+W_	0.618 ± 0.029	0.694 ± 0.001	0.657 ± 0.014	0.313 ± 0.029	0.719 ± 0.021
CNN_O+Z+W+E_	0.621 ± 0.030	0.694 ± 0.001	0.658 ± 0.015	0.315 ± 0.030	0.719 ± 0.019
CNN_O+Z+W+E+EG_	0.617 ± 0.039	0.694 ± 0.001	0.657 ± 0.019	0.311 ± 0.039	0.718 ± 0.022
Independent test					
CNN_O+Z+W_	0.578 ± 0.009	0.694 ± 0.000	0.638 ± 0.004	0.274 ± 0.009	0.704 ± 0.003
CNN_O+Z+W+E_	0.584 ± 0.012	0.694 ± 0.000	0.641 ± 0.006	0.279 ± 0.012	0.703 ± 0.002
CNN_O+Z+W+E+EG_	0.597 ± 0.022	0.694 ± 0.000	0.647 ± 0.011	0.292 ± 0.021	0.703 ± 0.002

### 3.4 Comparison of CNN-based models and stacking ensemble models

We compared the performances of the CNN-based models and the stacking ensemble models for each of the three datasets. We found no statistical difference between the CNN_OH_ model and these stacking ensemble models for each dataset (Figure 9 and Supplementary Figures S9, S10). The observation that the meta-classifiers perform similarly to the base classifier is consistent with the previous report for predicting bacterial Type IV secreted effectors, in which the meta-classifier and base classifier performed similarly (Xiong et al., 2018). It suggests that the base classifiers may have sufficient predictive ability, and the stacking ensemble architecture does not constantly improve prediction accuracy.

### 3.5 Construction of the online EdeepSADPr predictor

We developed an online prediction tool for predicting human pSADPr sites extensively from different conditions, dubbed EdeepSADPr. This tool consists of three models, each corresponding to the prediction from the SADPr dataset, the serine phosphorylation dataset or the human proteome. As the CNN_OH_ classifier had no less predictive performance than other methods, we selected this classifier to construct EdeepSADPr. The usage of this tool was described as follows. After the model selection, the input sequence with the fasta format would be uploaded. The prediction results were output in tabular form with five columns: sequence header, position, sequence, prediction score, and prediction category. The predicted results can also be downloaded as a data file. EdeepSADPr is accessible via http://edeepsadpr.bioinfogo.org/.

## 4 Conclusion

The main goal of this study is the development of a model to predict pSADPr sites based on protein sequence information and the investigation of pSADPr’s characteristics. We developed different deep-learning classifiers and used them as base classifiers to construct a few stacking-based ensemble models. We found that the base classifiers and the ensemble models had similar performances. The reason why the performance of the ensemble model was not improved is that there may not be much difference between the features used for model construction or the base models may not comprehensively cover the pSADPr’s characteristics. In the near future, we may integrate sequential information, structural information and evolutionary information to improve model performance (Xu et al., 2021). Additionally, the performance may be boosted by increasing the data amount and optimizing the model architecture (Zhu et al., 2022). Moreover, we found the characteristics of pSADPr sites, which may boost the understanding of this crosstalk. In summary, we developed the first classifier to predict human pSADPr sites and expect accurate prediction facilitate the discovery of new pSADPr sites. This architecture is applicable to the model construction for predicting other types of *in situ* crosstalks.

## Data Availability

The original contributions presented in the study are included in the article/Supplementary Material, further inquiries can be directed to the corresponding author.
