# Supplementary material for: EdeepSADPr: an extensive deep-learning architecture for prediction of the in situ crosstalks of serine phosphorylation and ADP-ribosylation
Source: Front Cell Dev Biol. 2023 Apr 28;11:1149535. doi: 10.3389/fcell.2023.1149535 (PMC10175571; doi:10.3389/fcell.2023.1149535)
Supplement: Supplementary file 1 [file DataSheet1.docx]

Supplementary Material

EdeepSADPr: An extensive deep-learning architecture for prediction of the in situ crosstalks of serine phosphorylation and ADP-ribosylation

Haoqiang Jiang^1,2†^, Shipeng Shang^1†^, Yutong Sha^1†^, Lin Zhang^3^, Ningning He^1^, and Lei Li^4,1*^

^1^ College of Basic Medicine, Qingdao University, Qingdao, China

^2^ Sino Genomics Technology Co., Ltd., Qingdao, China

^3^ College of Computer Science and Technology, Qingdao University, Qingdao, China

^4^ Faculty of Biomedical and Rehabilitation Engineering, University of Health and Rehabilitation Sciences, Qingdao, China

**
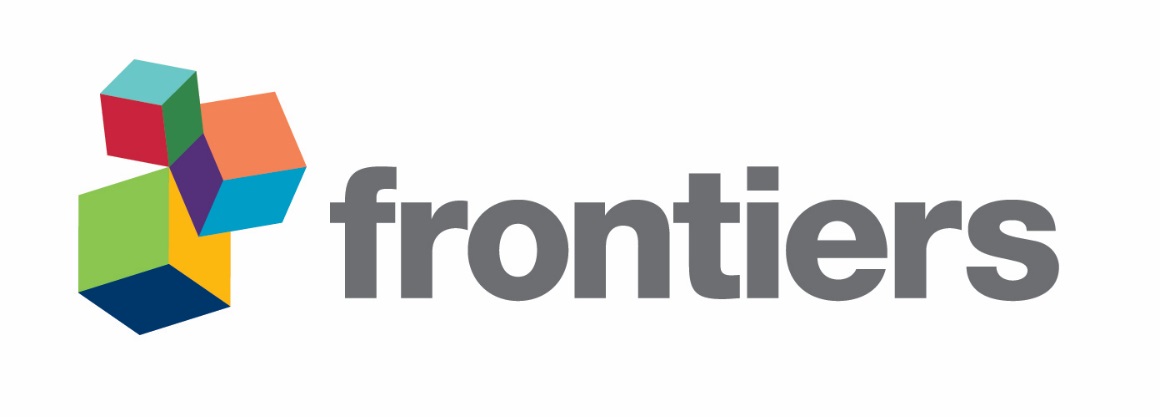
**

**Supplementary** **table 1. The average performance of different CNN-based classifiers in pSADPr-pS and pSADPr-UM datasets.**

|  | Classifier | SN | SP | ACC | MCC | AUC |
| --- | --- | --- | --- | --- | --- | --- |
| pSADPr-pS | Ten-fold Cross-validation |  |  |  |  |  |
|  | CNN_OH_ | 0.949±0.015 | 0.700±0.000 | 0.719±0.002 | 0.366±0.017 | 0.921±0.009 |
|  | CNN_ZSCALE_ | 0.944±0.017 | 0.700±0.000 | 0.719±0.003 | 0.363±0.020 | 0.919±0.010 |
|  | CNN_WE_ | 0.939±0.021 | 0.700±0.000 | 0.718±0.003 | 0.361±0.021 | 0.914±0.010 |
|  | CNN_EAAC_ | 0.906±0.025 | 0.700±0.000 | 0.716±0.003 | 0.343±0.023 | 0.894±0.011 |
|  | CNN_EGAAC_ | 0.850±0.025 | 0.700±0.000 | 0.711±0.003 | 0.312±0.022 | 0.860±0.020 |
|  | Independent test |  |  |  |  |  |
|  | CNN_OH_ | 0.936±0.005 | 0.700±0.000 | 0.718±0.000 | 0.359±0.003 | 0.914±0.003 |
|  | CNN_ZSCALE_ | 0.938±0.013 | 0.700±0.000 | 0.719±0.001 | 0.360±0.007 | 0.915±0.004 |
|  | CNN_WE_ | 0.931±0.020 | 0.700±0.000 | 0.718±0.002 | 0.356±0.011 | 0.905±0.008 |
|  | CNN_EAAC_ | 0.891±0.011 | 0.700±0.000 | 0.715±0.001 | 0.334±0.006 | 0.886±0.003 |
|  | CNN_EGAAC_ | 0.843±0.007 | 0.700±0.000 | 0.711±0.001 | 0.308±0.004 | 0.851±0.017 |
| pSADPr-UM | Ten-fold Cross-validation |  |  |  |  |  |
|  | CNN_OH_ | 0.981±0.006 | 0.700±0.000 | 0.710±0.001 | 0.270±0.010 | 0.953±0.003 |
|  | CNN_ZSCALE_ | 0.979±0.012 | 0.700±0.000 | 0.710±0.001 | 0.269±0.010 | 0.943±0.004 |
|  | CNN_WE_ | 0.979±0.007 | 0.700±0.000 | 0.710±0.001 | 0.269±0.010 | 0.942±0.004 |
|  | CNN_EAAC_ | 0.971±0.008 | 0.700±0.000 | 0.709±0.001 | 0.266±0.011 | 0.939±0.005 |
|  | CNN_EGAAC_ | 0.924±0.018 | 0.700±0.000 | 0.708±0.001 | 0.247±0.010 | 0.904±0.019 |
|  | Independent test |  |  |  |  |  |
|  | CNN_OH_ | 0.990±0.003 | 0.700±0.000 | 0.710±0.000 | 0.273±0.001 | 0.954±0.002 |
|  | CNN_ZSCALE_ | 0.991±0.006 | 0.700±0.000 | 0.710±0.000 | 0.274±0.002 | 0.943±0.003 |
|  | CNN_WE_ | 0.987±0.006 | 0.700±0.000 | 0.710±0.000 | 0.272±0.002 | 0.943±0.004 |
|  | CNN_EAAC_ | 0.987±0.007 | 0.700±0.000 | 0.710±0.000 | 0.272±0.003 | 0.944±0.002 |
|  | CNN_EGAAC_ | 0.937±0.012 | 0.700±0.000 | 0.708±0.000 | 0.253±0.005 | 0.911±0.017 |

Note: In ten-fold cross-validation, ten CNN-based models are constructed using ten different validation data sets. The ten models' average performance and standard deviation were calculated for the cross-validation and independent test datasets, respectively.

**Supplementary** **table 2. The performances of different stacking-based ensemble classifiers in pSADPr-pS and pSADPr-UM datasets**

|  | Classifier | SN | SP | ACC | MCC | AUC |
| --- | --- | --- | --- | --- | --- | --- |
| pSADPr-pS | Ten-fold Cross-validation |  |  |  |  |  |
|  | CNN_O+Z+W_ | 0.949±0.021 | 0.700±0.000 | 0.719±0.002 | 0.366±0.011 | 0.922±0.012 |
|  | CNN_O+Z+W+E_ | 0.948±0.020 | 0.700±0.000 | 0.719±0.002 | 0.366±0.011 | 0.923±0.012 |
|  | CNN_O+Z+W+E+EG_ | 0.947±0.023 | 0.700±0.000 | 0.719±0.002 | 0.365±0.012 | 0.923±0.013 |
|  | Independent test |  |  |  |  |  |
|  | CNN_O+Z+W_ | 0.940±0.004 | 0.700±0.000 | 0.718±0.000 | 0.361±0.002 | 0.917±0.001 |
|  | CNN_O+Z+W+E_ | 0.938±0.006 | 0.700±0.000 | 0.718±0.000 | 0.360±0.003 | 0.917±0.001 |
|  | CNN_O+Z+W+E+EG_ | 0.938±0.006 | 0.700±0.000 | 0.718±0.000 | 0.360±0.003 | 0.918±0.001 |
| pSADPr-UM | Ten-fold Cross-validation |  |  |  |  |  |
|  | CNN_O+Z+W_ | 0.982±0.008 | 0.700±0.000 | 0.710±0.000 | 0.270±0.003 | 0.952±0.003 |
|  | CNN_O+Z+W+E_ | 0.982±0.008 | 0.700±0.000 | 0.710±0.000 | 0.270±0.003 | 0.953±0.003 |
|  | CNN_O+Z+W+E+EG_ | 0.981±0.008 | 0.700±0.000 | 0.710±0.000 | 0.270±0.003 | 0.953±0.003 |
|  | Independent test |  |  |  |  |  |
|  | CNN_O+Z+W_ | 0.995±0.001 | 0.700±0.000 | 0.710±0.000 | 0.275±0.001 | 0.955±0.000 |
|  | CNN_O+Z+W+E_ | 0.993±0.002 | 0.700±0.000 | 0.710±0.000 | 0.275±0.001 | 0.957±0.000 |
|  | CNN_O+Z+W+E+EG_ | 0.993±0.002 | 0.700±0.000 | 0.710±0.000 | 0.275±0.001 | 0.957±0.000 |


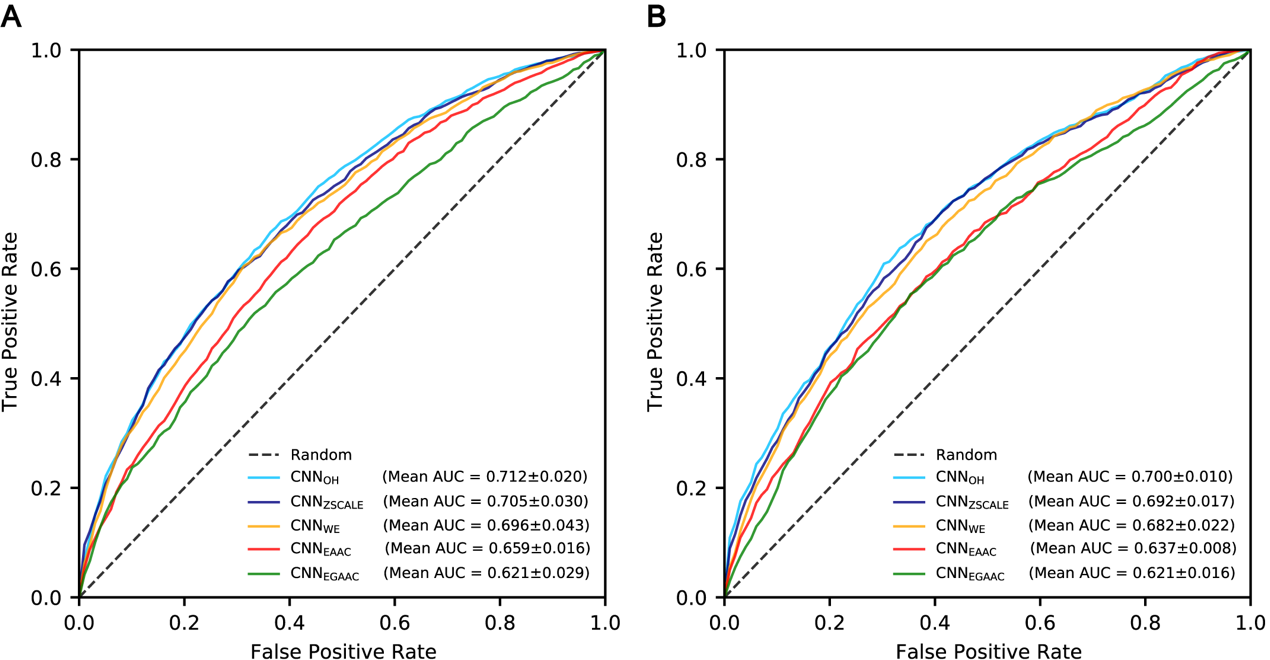


**Supplementary** **Figure 1. ROC curves of CNN-based models constructed using the pSADPr-SDAPr dataset in ten-fold cross-validation (A) and independent test(B).**


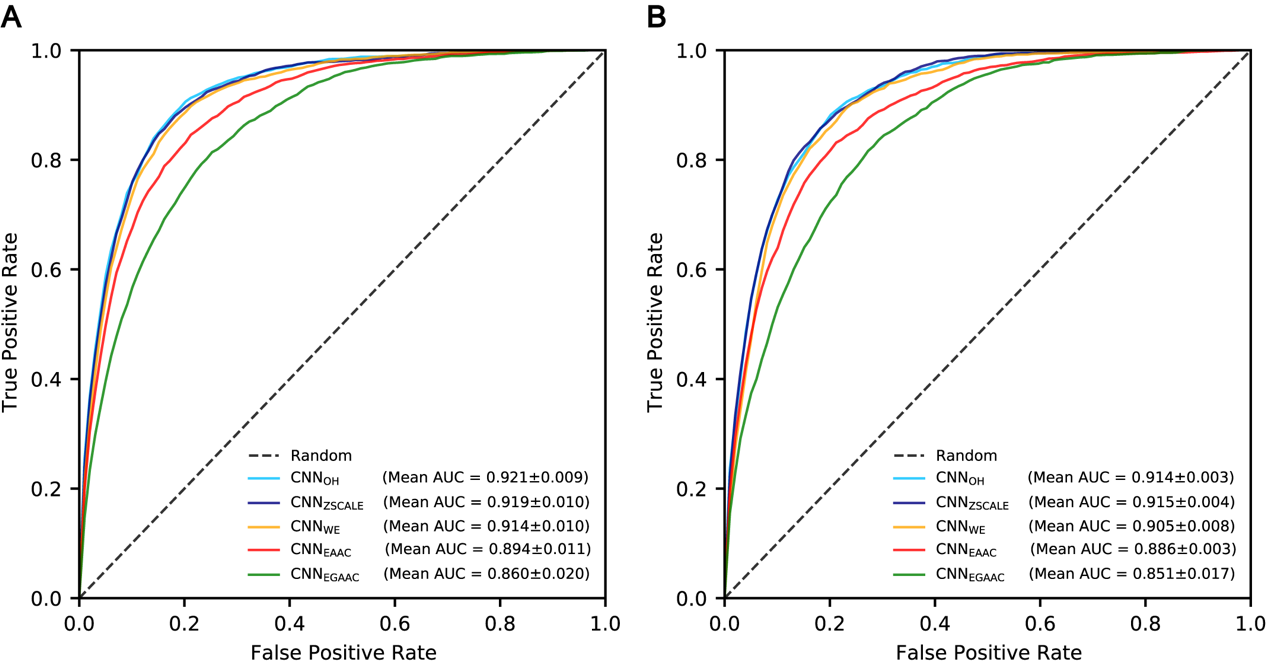


**Supplementary** **Figure 2**. **ROC curves of CNN-based classiﬁers built using the pSADPr-pS dataset in ten-fold cross-validation (A) and independent test (B).**


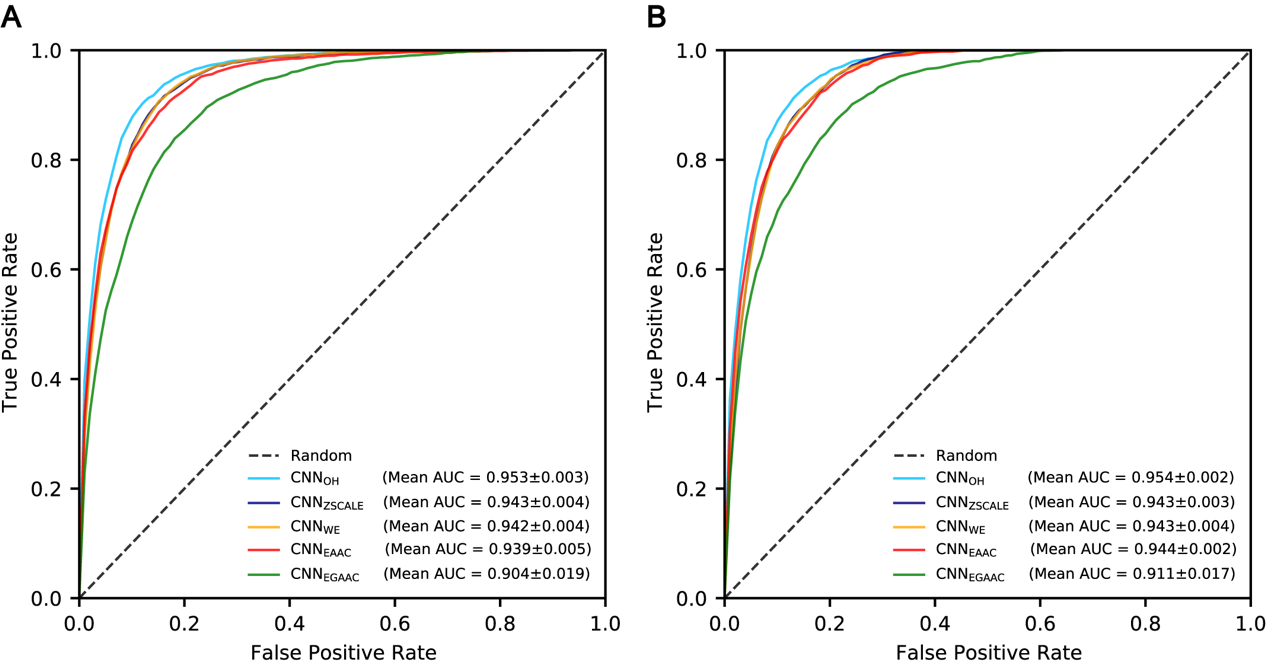


**Supplementary** **Figure 3**. **ROC curves of CNN-based classiﬁers constructed using the pSADPr-UM dataset in ten-fold cross-validation (A) and independent test (B).**


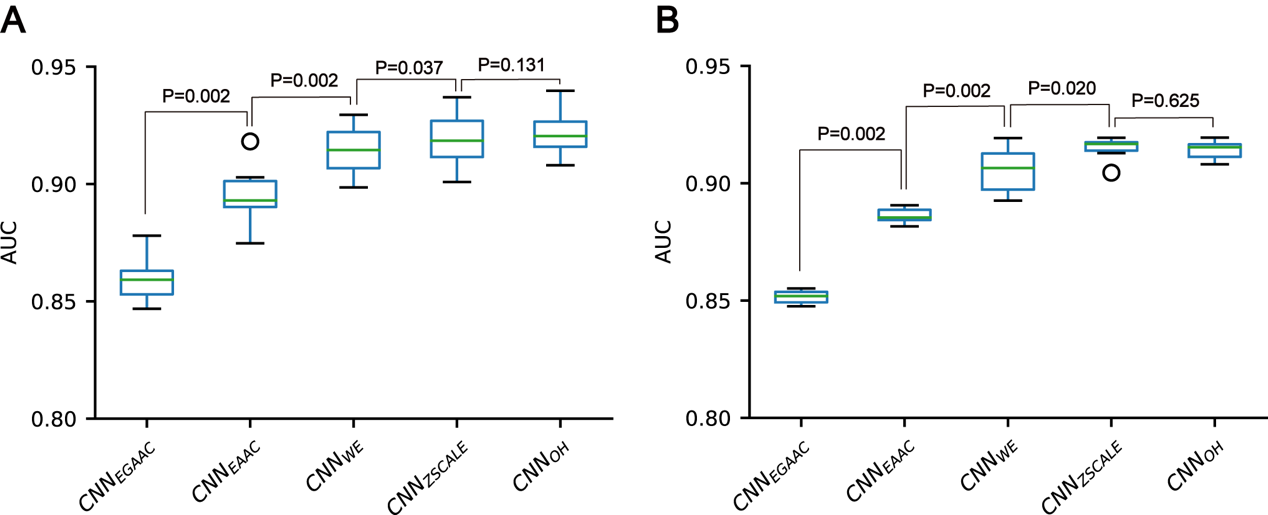


**Supplementary** **Figure 4.** **Performance comparisons between CNN-based classifiers for the pSADPr-pS dataset in the ten-fold cross-validation (A) and the independent test (B). P values were calculated using the two-sided Mann–Whitney U test.**


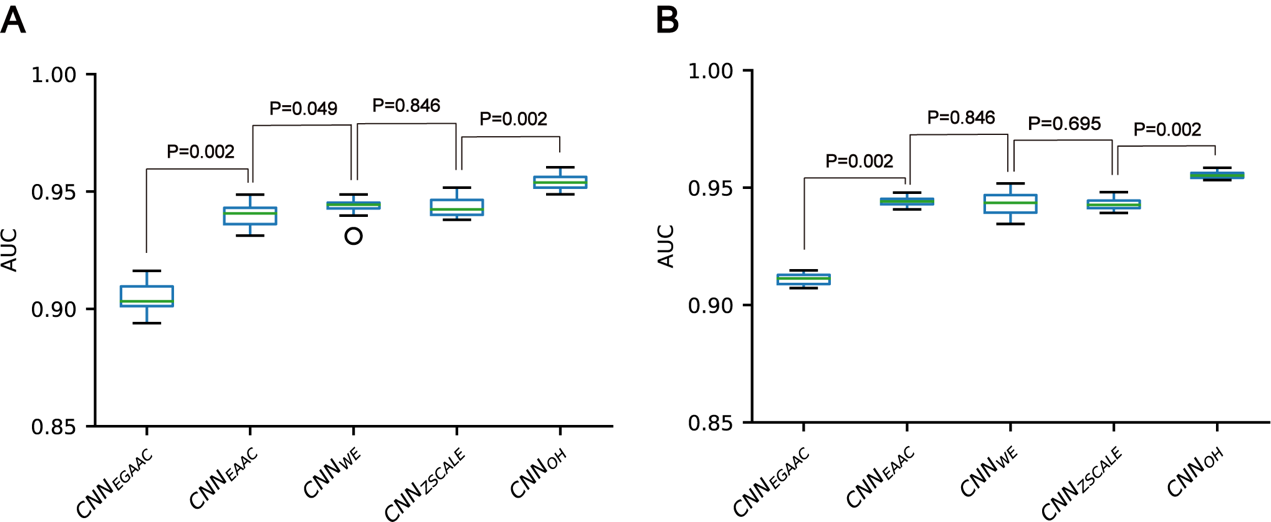


**Supplementary** **Figure 5**. **Performance comparisons between CNN-based classifiers for the pSADPr-UM dataset in the ten-fold cross-validation (A) and the independent test (B). P values were calculated using the two-sided Mann–Whitney U test.**


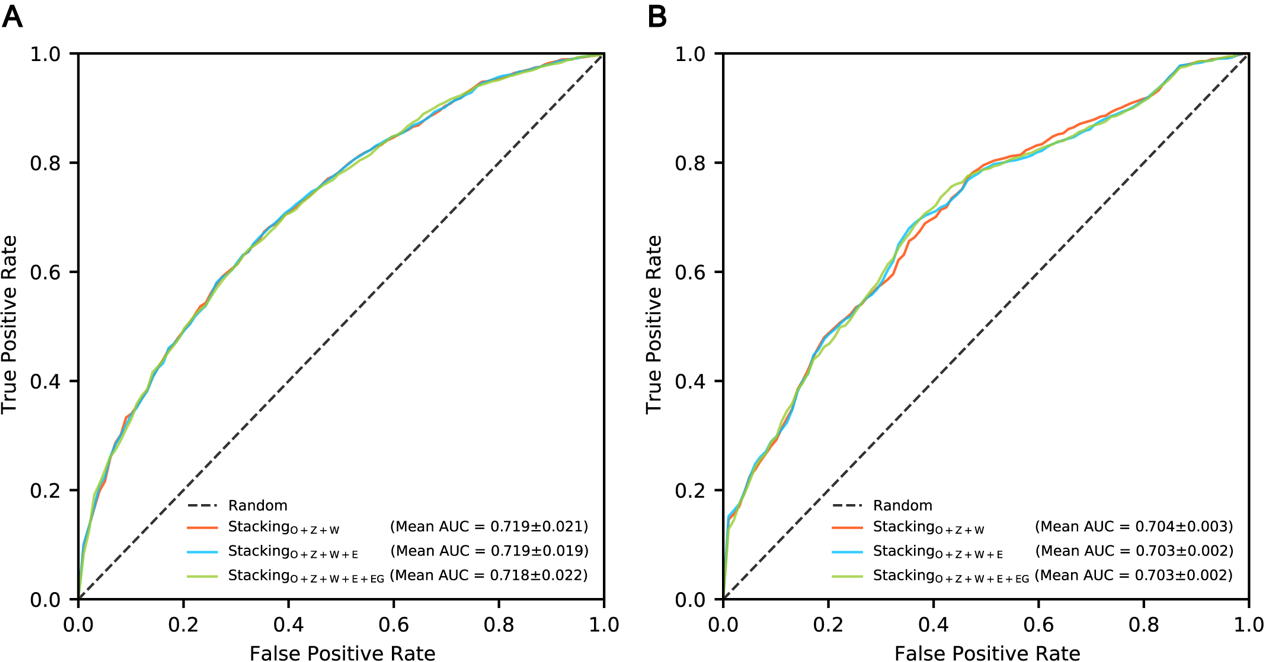


**Supplementary** **Figure 6. ROC curves of the stacking-based ensemble models constructed using the pSADPr-SADPr dataset in terms of ten-fold cross-validation (A) and independent test (B).**


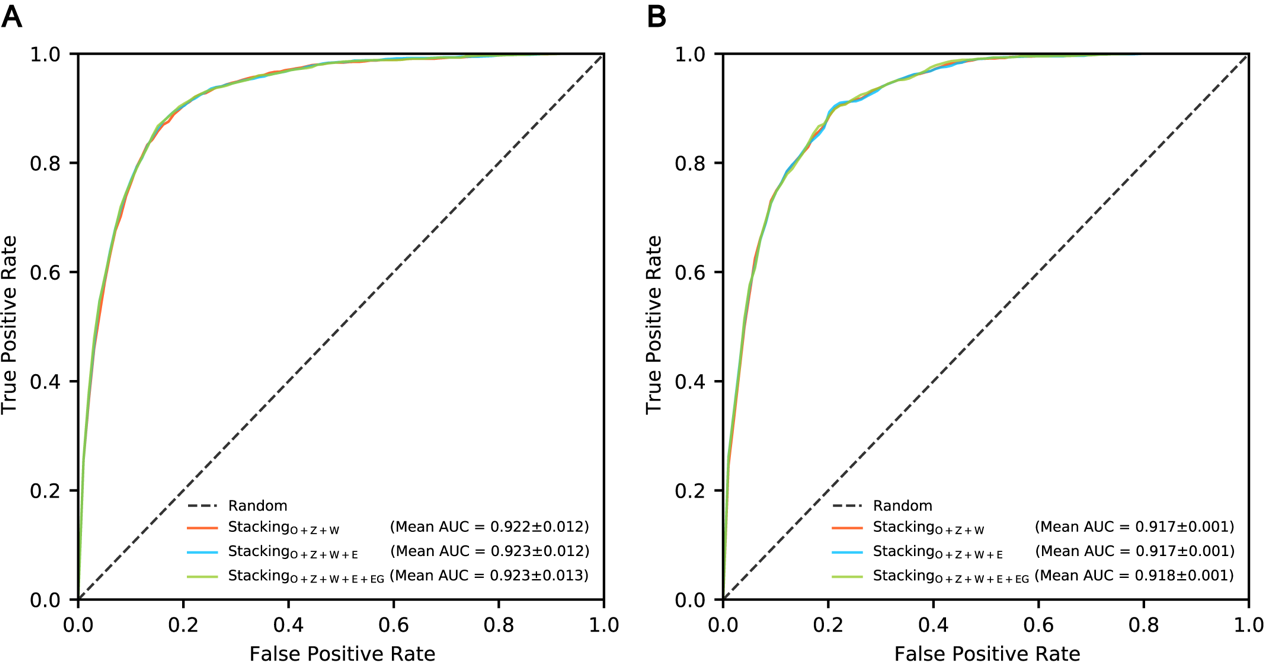


**Supplementary** **Figure 7. ROC curves of the stacking-based ensemble models constructed using the pSADPr-pS dataset in terms of ten-fold cross-validation (A) and independent test (B).**


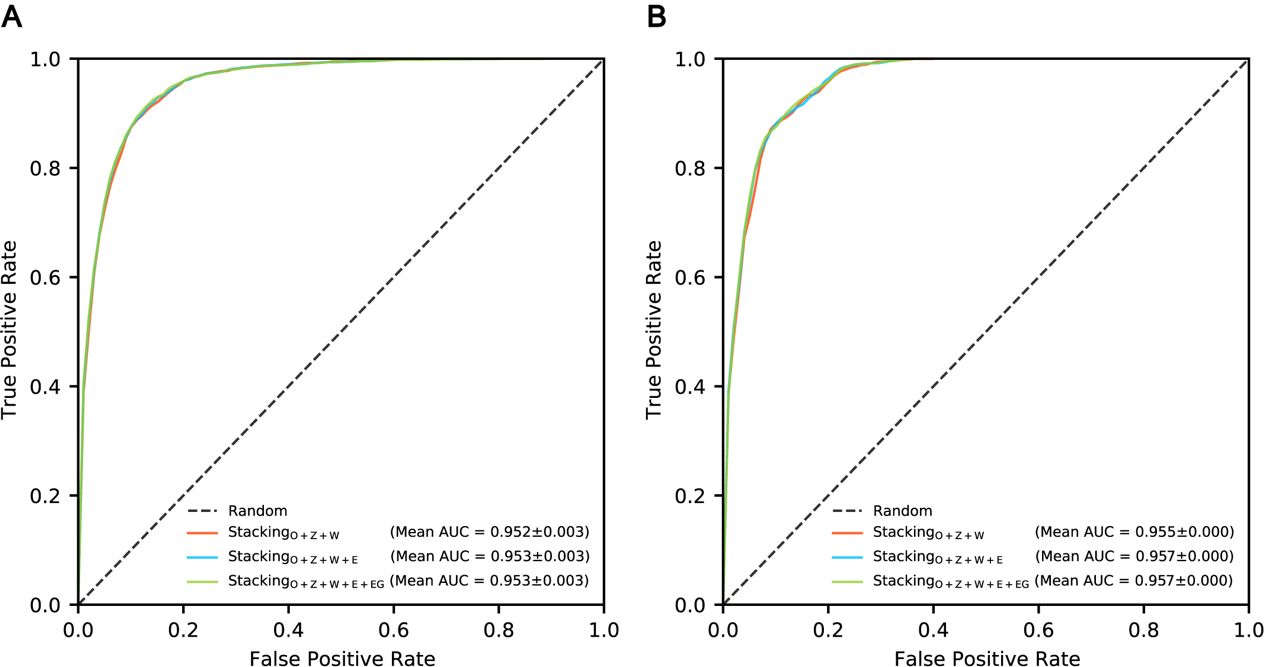


**Supplementary** **Figure 8. ROC curves of the stacking-based ensemble models constructed using the pSADPr-UM dataset in terms of ten-fold cross-validation (A) and independent test (B).**


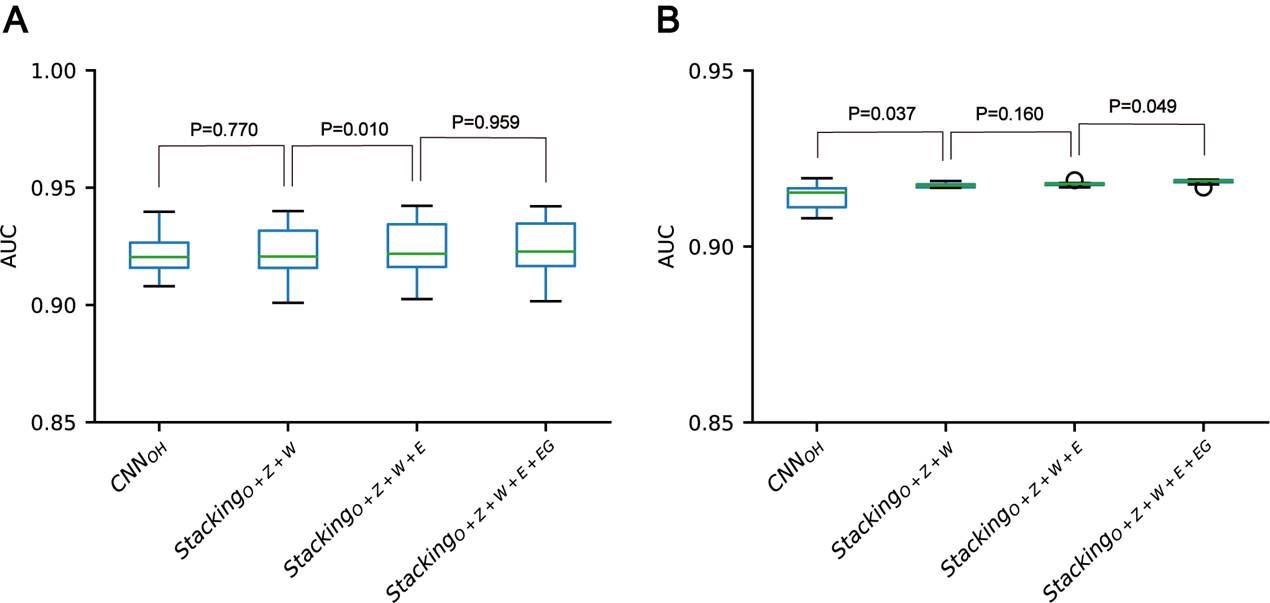


**Supplementary** **Figure 9**. **Performance comparisons between CNN-based and stacking-based ensemble classifiers for the pSADPr-pS dataset in the ten-fold cross-validation (A) and the independent test (B). P values were calculated using the two-sided Mann–Whitney U test.**


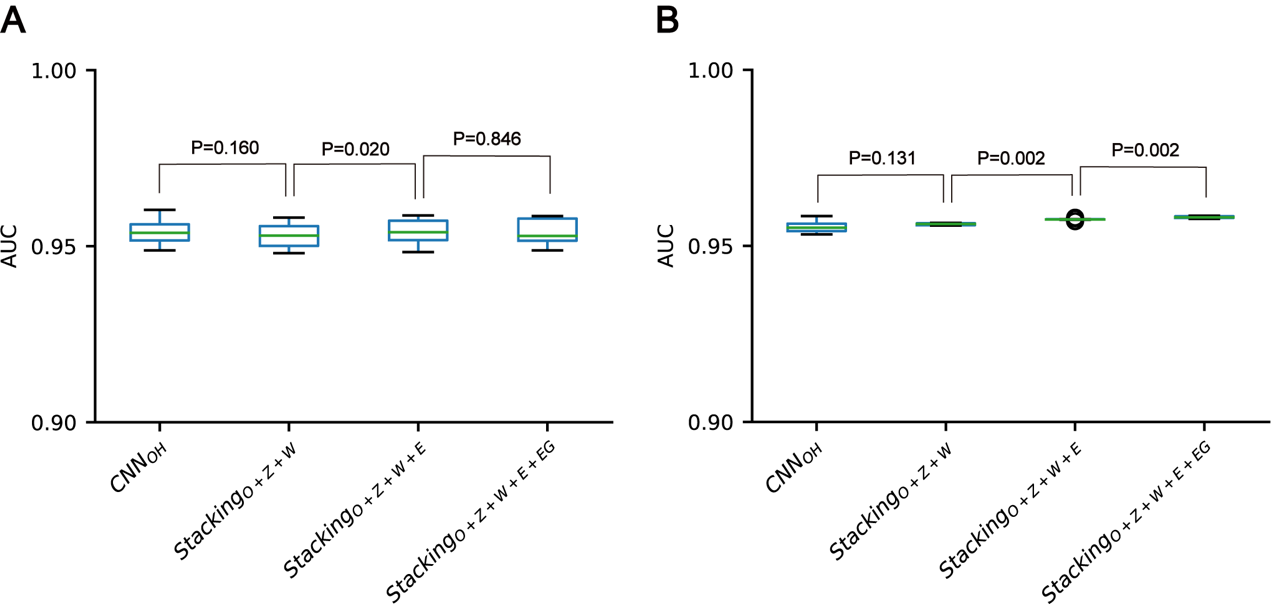


**Supplementary** **Figure** **10**. **Performance comparisons between CNN-based and stacking-based ensemble classifiers for the pSADPr-UM dataset in the ten-fold cross-validation (A) and the independent test (B). P values were calculated using the two-sided Mann–Whitney U test.**
